# Autophagy-dependent modulation of ER calcium release drives KCNMA1/BKCa signaling and seizure susceptibility

**DOI:** 10.1080/15548627.2025.2580436

**Published:** 2025-11-10

**Authors:** Gaga Kochlamazashvili, Marijn Kuijpers

**Affiliations:** a Department Molecular Pharmacology and Cell Biology, Leibniz-Forschungsinstitut für Molekulare Pharmakologie (FMP), Berlin, Germany; b Donders Institute for Brain, Cognition and Behaviour and Faculty of Science, Radboud University, Nijmegen, The Netherlands

**Keywords:** Autophagy, calcium, endoplasmic reticulum, ERphagy, axon, excitability, epilepsy, BKCa, ryanodine receptor, neuron

## Abstract

Macroautophagy/autophagy is best known for its role in maintaining cellular homeostasis through degradation of damaged proteins and organelles. In neurons, autophagy also contributes to the regulation of activity by adjusting the availability of cellular components to physiological demand. In a recent study, we show that autophagy shapes neuronal excitability by restraining a calcium-dependent pathway that couples endoplasmic reticulum calcium release to KCNMA1/BKCa activity at the plasma membrane. When autophagy is lost, this pathway is enhanced, and seizure susceptibility increases.

Neurons communicate with each other via electrical signals called action potentials, which are brief changes in the electrical potential across a membrane. An action potential is generated at the axon initial segment and propagates along its length. When it reaches the axon terminal, or presynapse, it triggers the release of neurotransmitters, that are subsequently picked up by receptors, e.g., on the dendrite of a postsynaptic neuron. The ability of a neuron to generate action potentials in response to stimuli, known as intrinsic excitability, depends primarily on voltage-gated sodium (Na_V_) and potassium (K_V_) channels in the plasma membrane. An action potential starts when a Na_v_ opens, letting Na^+^ rush in and rapidly depolarize the membrane. It ends as a K_v_ opens, allowing K^+^ efflux to repolarize the membrane. Disruptions in the function, expression, or localization of these channels can lead to abnormal electrical activity and underlie disorders of excitability, including epilepsy. While neuronal autophagy is most famously known for its ability to clear aggregation-prone proteins, thereby counteracting neurodegeneration, recent work also highlights roles for autophagy in the regulation of neurotransmission and behavior. Autophagy can operate locally in different neuronal compartments such as axons, dendrites and synapses, where it can shape specialized functions, ranging from neurotransmitter release to receptor trafficking and excitability. Conversely, altered autophagy can contribute to the occurrence of epilepsy. This underscores that autophagy contributes not only to cellular quality control but also to dynamic regulation of neuronal signaling. However, little is known about the specific cellular substrates and pathways that brain autophagy uses to control these dynamic alterations.

To address this, we used conditional *atg5* knockout mice (*atg5* cKO) in which *Atg5*, i.e., the gene encoding an essential component of the LC3-lipidation machinery, is deleted in excitatory neurons, thereby hampering efficient neuronal autophagy [1]. Two observations motivated a focus on excitability. First, *atg5* cKO mice show severely impaired postnatal viability. Second, ATG5 loss makes neural circuits easier to drive to a maximal response. These findings prompted us to test whether loss of autophagy increases intrinsic excitability. In hippocampal brain slices we used kainate, a standard excitatory compound, to drive neuronal network activity and observe a marked increase in epileptiform bursts in *atg5* cKO brains. To gain further insight into the basis of this lowered seizure threshold, we recorded stimulus-evoked electrical activity from transected axons in brain slices, thereby isolating axonal properties from cell bodies and synapses. These recordings show that axons require less stimulation to fire action potentials, pinpointing axonal hyperexcitability as a driver of the increased seizure-like bursting in *atg5* cKO slices.

Guided by these results, we focused on the endoplasmic reticulum (ER), the principal internal calcium store in neurons. Prior work showed that loss of neuronal autophagy causes axonal ER to accumulate and increases calcium efflux through RYR (ryanodine receptor) proteins, large calcium-release channels in the ER membrane (Figure 1). In the presynapse, this change in ER calcium homeostasis facilitates the fusion of neurotransmitter-filled vesicles with the plasma membrane, a process dependent on local calcium concentrations. Using electrophysiological recordings in *atg5* cKO brain slices, we now can show that pharmacological blockade of RYRs rescues this increase in presynaptic neurotransmitter release, demonstrating that aberrant RYR-mediated calcium signaling underlies the enhanced presynaptic activity. Strikingly, RYR inhibition also normalizes axonal excitability and restores the elevated seizure susceptibility, identifying excessive ER calcium release as a central driver of the phenotype.

**Figure 1.**
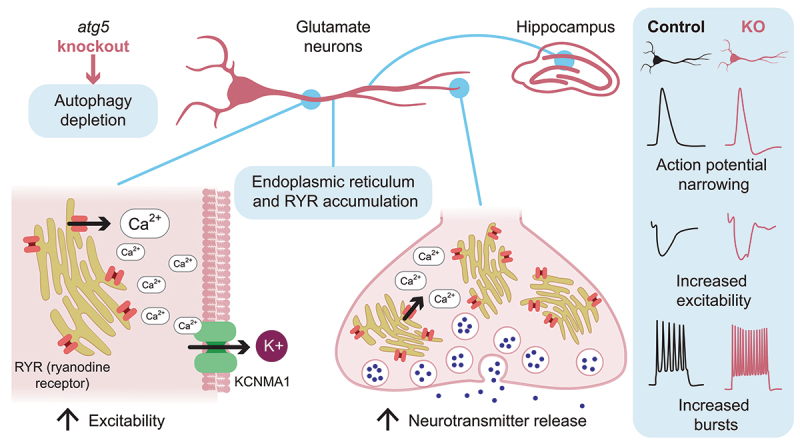
Excessive endoplasmic reticulum (ER) calcium release emerges as a central consequence of impaired autophagy. Conditional deletion of *Atg5* in excitatory glutamatergic neurons abolishes autophagy and leads to accumulation of ER and RYRs (ryanodine receptors), specifically in the axon. In presynaptic terminals, the associated change in calcium homeostasis facilitates vesicle fusion and increases neurotransmitter release probability. In axons, calcium leakage through RYRs activates large-conductance calcium-activated potassium (KCNMA1) channels. KCNMA1 activity accelerates repolarization, narrows action potentials, and increases the capacity for repetitive firing, thereby promoting neuronal excitability. This axonal mechanism lowers the seizure threshold in *atg5* cKO mice.

We next asked how RYR-mediated calcium leakage could potentially influence axonal excitability. An important clue came when we took a closer look at the characteristics and shape of the action potentials. Using whole-cell patch clamp recordings we can gain electrical access to a single neuron’s interior and study the properties of action potentials. These recordings show an increased negative slope, or decreased action potential half-width, in *atg5* cKO neurons, suggestive of a facilitated repolarizing K_V_ channel function. Strikingly, this change is also reversed by RYR inhibition, implicating RYR-dependent calcium release in shaping action potentials and axonal excitability.

Combining these observations we focused on a particular Kv channel – the large conductance calcium-activated potassium channel KCNMA1/BKCa. KCNMA1 channels are expressed widely and play important roles in many processes, including the repolarization of the neuronal membrane potential. They are synergistically activated by membrane depolarization and by increases in intracellular calcium and are therefore spatially and functionally coupled to calcium sources such as ER-localized RYRs. We hypothesized that enhanced RYR-mediated calcium release leads to increased KCNMA1 activity, contributing to the observed hyperexcitability phenotype. Consistent with this idea, electrophysiological measurements show that inhibition of KCNMA1 rescues the reduced action potential half-width of *atg5* cKO neurons, similar to the effect of RYR inhibition. Moreover, blocking KCNMA1 channels significantly reduces kainate-induced epileptic discharges in *atg5* cKO mice, as well as reducing the axonal hyperexcitability. In contrast, inhibition of KCNMA1 channels does not alter the elevated presynaptic release of neurotransmitters, indicating that this phenotype is KCNMA1 independent.

Loss of autophagy causes axonal ER to accumulate, and our findings show that this accumulated ER perturbs neuronal signaling through two distinct RYR-centered mechanisms. First, excessive ER calcium release elevates presynaptic vesicle fusion probability, thereby strengthening synaptic transmission. Second, the same aberrant calcium release activates KCNMA1 channels, which sharpen action potential repolarization and increase axon excitability (Figure 1). Importantly, this gain in KCNMA1 function is not due to altered channel abundance or localization, because quantitative proteomics and immunoblotting revealed no changes in KCNMA1 or other Kv channel levels. Immunolabeling further shows that while RYRs accumulate in axons, KCNMA1 distribution remains unchanged and partially overlaps with RYRs, consistent with their functional coupling. Taken together, excessive ER calcium release emerges as a central consequence of impaired autophagy. A key question now is how this regulatory role of autophagy plays out in normal neuronal physiology. Axonal calcium is central for transmitter release, shaping of action potentials, and synaptic plasticity, and autophagy could be part of the machinery that adjusts these processes in everyday conditions. The link between autophagy, or ERphagy, and fast electrical signaling offers a new way to think about how neurons keep their activity in check, and how loss of this control can increase seizure susceptibility.
